# The preventive effects of *Lactobacillus casei* 03 on *Escherichia coli*-induced mastitis in vitro and in vivo

**DOI:** 10.1186/s12950-024-00378-x

**Published:** 2024-02-23

**Authors:** Ke Li, Ming Yang, Mengyue Tian, Li Jia, Yinghao Wu, Jinliang Du, Lining Yuan, Lianmin Li, Yuzhong Ma

**Affiliations:** 1 College of Veterinary Medicine, Shandong Agricultural University, 271018 Taian, Shandong China; 2 College of Veterinary Medicine, Hebei Agricultural University, 2596 Lekai South Street, 071001 Baoding, Hebei China; 3 College of Life Science and Food Engineering, Hebei University of Engineering, 056038 Handan, Hebei China; 4 Key Laboratory of Freshwater Fisheries and Germplasm Resources Utilization, Freshwater Fisheries Research Center, Ministry of Agriculture, Chinese Academy of Fishery Sciences, 214081 Wuxi, China

**Keywords:** Mastitis, *Lactobacillus casei*, Inflammation, Signal path, In vitro and in vivo

## Abstract

**Background:**

*Lactobacillus casei* possesses many kinds of bioactivities, such as anti-inflammation and anti-oxidant, and has been applied to treating multiple inflammatory diseases. However, its role in mastitis prevention has remained ambiguous.

**Methods:**

This study aimed to examine the mechanisms underlying the preventive effects of *L. casei* 03 against *E. coli*- mastitis utilizing bovine mammary epithelial cells (**BMECs**) and a mouse model.

**Results:**

In vitro assays revealed pretreatment with *L. casei* 03 reduced the apoptotic ratio and the mRNA expression levels of *IL1β*, *IL6* and *TNFα* and suppressed phosphorylation of p65, IκBα, p38, JNK and ERK in the NF-κB signaling pathway and MAPK signaling pathway. Furthermore, in vivo tests indicated that intramammary infusion of *L. casei* 03 relieved pathological changes, reduced the secretion of IL1β, IL6 and TNFα and MPO activity in the mouse mastitis model.

**Conclusions:**

These data suggest that *L. casei* 03 exerts protective effects against *E. coli*-induced mastitis in vitro and in vivo and may hold promise as a novel agent for the prevention and treatment of mastitis.

**Supplementary Information:**

The online version contains supplementary material available at 10.1186/s12950-024-00378-x.

## Background

Mastitis is a common disease associated with dairy cattle and causes pathological alterations in the mammary gland tissue [1]. It is characterized by high economic losses because of high morbidity and impaired production [2]. Some studies revealed that mastitis in dairy cows could affect the physiological status of newborn calves during pregnancy [3], and the growth and the acquisition of immune antibodies in calves may be influenced due to drinking low-quality milk from mastitis udders [4]. Also, the pathogen can be vertically transmitted to calves via contaminated milk and colostrum, causing gastrointestinal diseases and even death [5].

Dairy cow mastitis is mainly caused by pathogen infection [6]. A recent report shows that *Escherichia coli* is the main pathogen of acute mastitis in dairy farming and it is generally considered an environmental pathogen that can enter the mammary gland through the teat canal under appropriate conditions [7, 8]. Invasive *E. coli* release endotoxins such as LPS and induce strong responses from the cows’ immune systems, which in turn activates the blood clotting system, leading to local microcirculation disturbances and tissue damage [9]. Currently, antimicrobials have been widely used to prevent and control mastitis and other bacterial diseases in intensive food animal production [10]. Reliance on antibiotics inevitably leads to the onset of antibiotic resistant bacterial strains, which threaten animal production and human health.

As a possible alternative to antibiotics, probiotics caught wide attention and have elicited numerous related research in recent years [11]. One such probiotic, *Lactobacillus casei*, is a commensal bacterium generally found in human and animal intestines which can enhance animal immune capacity and promote animal growth and development [12]. With the deepening of research, more and more researchers have begun to focus on the potential anti-inflammatory effects of *L. casei*. Zhang et al. [13] showed that *L. casei* Zhang might prevent experimental colitis and rapamycin-induced inflammation in the mice. Haro et al. [14] demonstrated that *L. casei* could modulate inflammation-coagulation interactions in an experimental model of pneumococcal pneumonia. The study by Chen et al. [2] suggested that *L. plantarum* KLDS 1.0344 showed good antibacterial properties and may be developed into a probiotic formulation for inflammatory disease control and prevention. These findings herald that *L. casei* may be an effective prevention strategy for dairy cow mastitis. However, the exact mechanism of its action remains unclear. In this study, we investigated the preventive effect of *Lactobacillus casei* 03 on the *E. coli* -induced inflammatory model and its mechanism of action in vitro and in vivo, in order to provide a theoretical basis for developing novel drugs for mastitis prevention and treatment in cows.

## Materials and methods

### Bacterial and cultural conditions

The *Lactobacillus casei* 03 strain obtained from American Type Culture Collection (**ATCC393**; Manassas, VA, USA) and was cultivated in de Man, Rogosa, and Sharpe (**MRS**) broth (Aobox, Beijing, China) under microaerobic conditions at 37℃ for 48 h. *Escherichia coli* O111:K58 (CVCC1450, provided by China Constitute of Veterinary Drug Centre, Beijing, China) was grown overnight in LB medium (Aobox, Beijing, China) with shaking at 37℃. Bacterial colony counts (**CFU**) were calculated and recorded after three generations.

### Cells culture and treatment

Bovine mammary epithelial cells (**BMECs**) were provided by Animal Clinical Laboratory of Hebei Agricultural University (Baoding, China) and grown in Dulbecco’s modified Eagle’s medium/Ham’s F12 nutrient mixture (DMEM/F12, Gibco, Grand Island, USA) supplemented with 15% FBS (Gibco), 0.1% hydrocortisone (Sigma-Aldrich, MO, USA), 100 U/mL penicillin-streptomycin (Solarbio, Beijing, China) and 0.025 M HEPES (Solarbio) at 37℃ with 5% CO_2_. When cells were 70–80% confluence, they were treated as follows in six-well plates: the CON group (DMEM/F12), the ECOL group (10^7^ CFU/mL *E. coli*), pretreated with *L. casei* 03 (10^4^, 10^5^ and 10^6^ CFU/mL) for 3 h before the addition of *E. coli*, the LC group (10^6^ CFU/mL *L. casei* 03). Eight hours after *E. coli* (10^7^ CFU/mL) infection, cells were washed with PBS and collected for subsequent assays.

### Cell viability assay

The effect of different doses of *L. casei* 03 on BMECs viability was determined using the CCK-8 assay. In brief, cells were plated in 96-well plates and cultured to approximately 80% confluence. Subsequently, the cells were treated with various concentrations of *L. casei* 03 (10^3^ to 10^8^ CFU/mL) for 3 h and CCK-8 reagent was added to each well to incubate at 37℃ for another 2 h. The absorbance of the wells was read at 450 nm on a microplate reader.

### Cell immunofluorescence staining

Treated cells were stained using the Annexin V-FITC/Propidium Iodide Apoptosis Detection Kit (#C1062M, Beyotime Shanghai, China) according to the manufacturer’s instructions. Images were visualized using a fluorescence microscope. Five visual fields were selected randomly for cell counting. Annexin V-positive cells were labeled as the apoptotic cells and the apoptotic rate (%) was calculated as the number of apoptotic cells/total number of cells×100%.

### qRT–PCR analysis

Total RNA extraction was performed using Ultrapure RNA extraction kit (CWBio, Beijing, China). The purity, concentration and integrity of the RNA samples were assessed by spectrophotometry and electrophoresis. Subsequently, RNA samples were reverse transcribed into cDNA using reverse transcription kit (US Everbright Inc, CA, USA) for quantitative Real-Time PCR (qRT-PCR) analyses. The reaction program was 95℃ for 2 min, 95℃ for 15 s, 58 ℃ for 30 s, and 72℃ for 30 s for 40 cycles. The expression level of target genes was normalized to *GAPDH* and *β-actin* and calculated with the 2^−ΔΔCt^ method. The primer sequences were given in Table 1.

**Table 1:** Sequence of primers used in qRT-PCR

Gene	Direction	Primer sequence (5’-3’)	Product sizes (bp)
*IL1β*	F	GAGCCTGTCATCTTCGAAACG	55
	R	GCACGGGTGCGTCACA	
*IL6*	F	GCTGAATCTTCCAAAAATGGAGG	200
	R	GCTTCAGGATCTGGATCAGTG	
*TNFα*	F	TCCAGAAGTTGCTTGTGCCT	144
	R	CAGAGGGCTGTTGATGGAGG	
*GAPDH*	F	CACCCTCAAGATTGTCAGCA	103
	R	GGTCATAAGTCCCTCCACGA	
*β-actin*	F	CTGTGCTGTCCCTGTATGCC	222
	R	TGTCACGGACGATTTCCCGCT	

### Western blot analysis

The total protein from cells was extracted using RIPA lysis buffer (Solarbio, Beijing, China) and quantified with the BCA protein assay kit (Solarbio, Beijing, China). Protein samples (25 µg) were separated by 10% SDS-PAGE, electrotransferred onto a nitrocellulose membrane and blocked with 5% milk. The membranes were incubated against NF-κB p65 (1:1000), NF-κB phospho-p65 (1:1000), phospho-IκBα (1:500) and β-actin (1:1000) from Bioss Biotech Limited Company (Beijing, China) and antibodies against p38 (1:1000), phospho-p38 (1:1000), ERK (1:1000), phospho-ERK (1:2000), JNK (1:1000), phospho-JNK (1:1000) and IκBα (1:1000) from Cell Signaling Technology (MA, USA) at 4℃ with primary antibodies overnight and then with the corresponding secondary antibodies (1:2000, Zhongshan Golden Bridge, Beijing, China) for 1 h. Finally, the blots were stained using BCIP/NBT color development kit (Solarbio, Beijing, China) and analyzed for grayscale values using Image J software.

### Animals and experiment design

SPF-grade male and female KM mice (6–8 weeks old) were purchased from Liaoning Changsheng Biotechnology Corporation (Benxi, China). After 3 days of adaptive diet feeding, male and female mice (1:3 ratio) were placed in the same cage until the female mice became pregnant and were able to drink and eat freely during the experiment. The lactating mice were randomly divided into four groups: the CON group (PBS), the ECOL group (10^7^ CFU/100 µL), the LC + ECOL group: pretreated with *L. casei* 03 (10^6^ CFU/100 µL) for 3 h before the addition of *E. coli* and the LC group (10^6^ CFU/100 µL *L. casei* 03). Mice were anesthetized by ether and their fourth pair of nipples were sterilized with 75% ethanol. Then, the mice were injected with 100 µL bacterium or PBS (50 µL/side) into their nipples with 32G needle. After 24 h of infection, mice were sacrificed and the mammary gland tissues were collected for subsequent experiments. Animal assays were approved by the Animal Ethics Committee of Hebei Agricultural University (Protocol number 2,020,044).

### Histopathologic analysis

Tissue samples were fixed in 4%-buffered paraformaldehyde solution, dehydrated with alcohol, and embedded in paraffin. Subsequently, paraffin-embedded tissues were cut into 5 μm sections, which were routinely stained with HE, followed by observation under a light microscope. Histological scores were performed according to the criteria previously described [15]. Briefly, the scores were graded on edema, inflammatory cell infiltration, hyperemia and necrosis. Scores range from 1 to 5, with higher scores indicating greater inflammation.

### MPO activity determination and cytokines analysis

Mammary gland tissues were homogenized and centrifuged (2500 rpm for 10 min) to collect supernatants. The MPO activity in mammary gland tissues was detected according to the manufacturer’s instructions (Nanjing Jiancheng Bioengineering Institute, Nanjing, China). Cytokine concentrations in supernatants were measured using ELISA kits from Shanghai Enzyme-linked Biotechnology (Shanghai, China).

### Statistical analysis

The data were presented as means ± standard error of the mean (**SEM**). One-way analysis of variance and Tukey’s or Dunnett’s T3 test were used for the comparison among groups. *P* < 0.05 were considered significant.

## Results

### Effects of *L. casei* 03 on the viability of BMECs

**Fig. 1:**
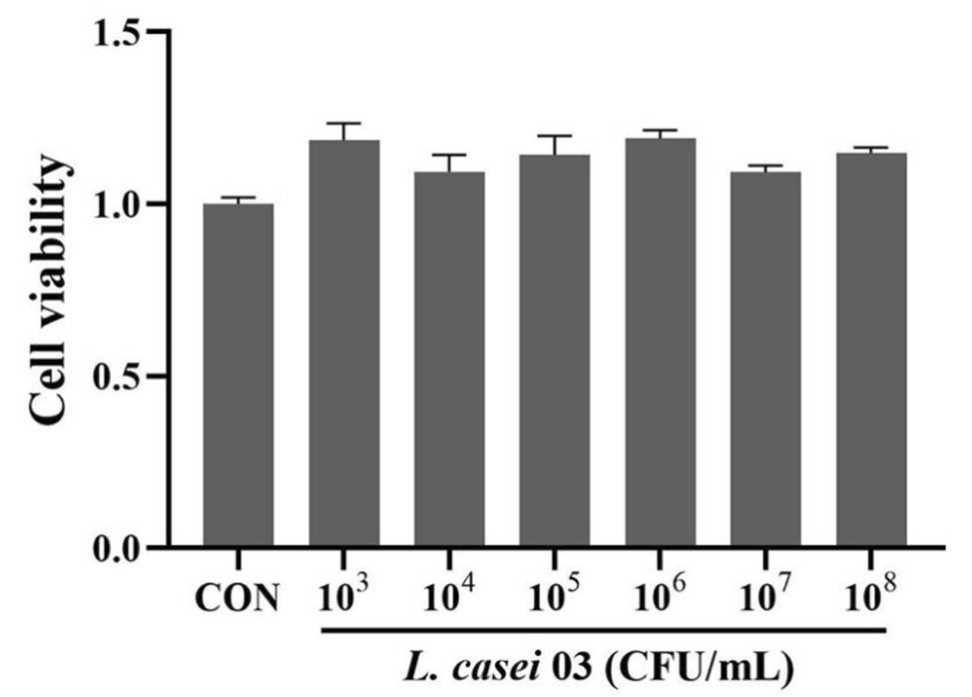
Effects of *L. casei* 03 on the cell viability in BMECs. Cells were cultured with different concentrations of *L. casei* 03 (10^3^ to 10^8^) for 3 h by CCK-8 assay. The data were presented as the means ± SEM of five independent experiments

The CCK-8 assay showed that BMECs viability was not affected upon treatment with 10^3^ to 10^8^ CFU/mL *L. casei* 03 (Fig. 1). The concentrations (10^4^, 10^5^ and 10^6^ CFU/mL) of *L. casei* 03 were accordingly selected for subsequent experiments.

### Effects of *L. casei* 03 on BMECs apoptosis

**Fig. 2:**
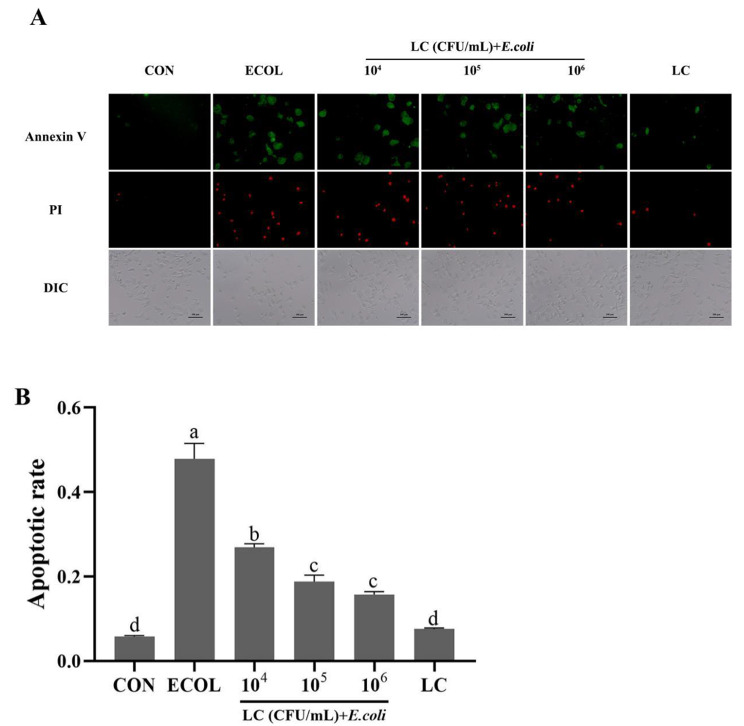
*L. casei* 03 inhibited BMECs apoptosis induced by *E. coli*. (**A**) Apoptosis was evaluated by measuring Annexin/PI fluorescent staining. Green, Annexin V-positive; Red, PI-positive; DIC, ordinary light; scale bar: 200 μm. (**B**) The apoptotic rate was quantified by counting the green-positive cells. Groups with different letters above the bar indicate significant differences (*P* < 0.05). These same conventions were used in subsequent figures

The cell apoptosis was analyzed using Annexin-V/PI double staining. As shown in Fig. 2A, B and E. *coli* treatment apparently increased the rate of Annexin-V positive cells compared with that in the CON group (*P* < 0.05). In contrast, pretreatment with different doses of *L. casei* 03 significantly reduced the rate of apoptotic cells (*P* < 0.05).

### Effects of *L. casei* 03 on the mRNA expression of pro-inflammatory genes in *E. coli*-induced BMECs

**Fig. 3:**
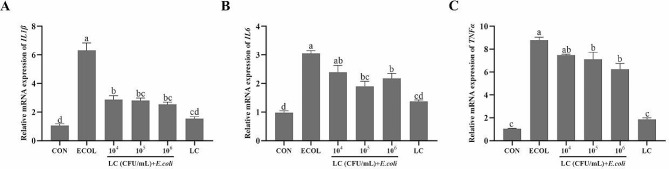
Effects of *L. casei* 03 on the mRNA expression of pro-inflammatory cytokines in *E. coli*-induced BMECs. The mRNA expression levels of *IL1β*, *IL6* and *TNFα* (**A-C**) in *E. coli*-induced BMECs were detected by qRT-PCR. Values were obtained from three independent experiments

The mRNA expression levels of *IL1β*, *IL6* and *TNF*α were detected by qRT-PCR (Fig. 3A-C). *E. coli* challenge caused a significant increase the expression levels of three pro-inflammatory genes compared with those in the CON group (*P* < 0.05). Pretreatment with different doses of *L. casei* 03 inhibited the expression in levels of *IL1β*, *IL6* and *TNFα* to varying degrees.

### Effects of *L. casei* 03 on the protein expression of the NF-κB and MAPK signaling pathways in *E. coli*-induced BMECs

**Fig. 4:**
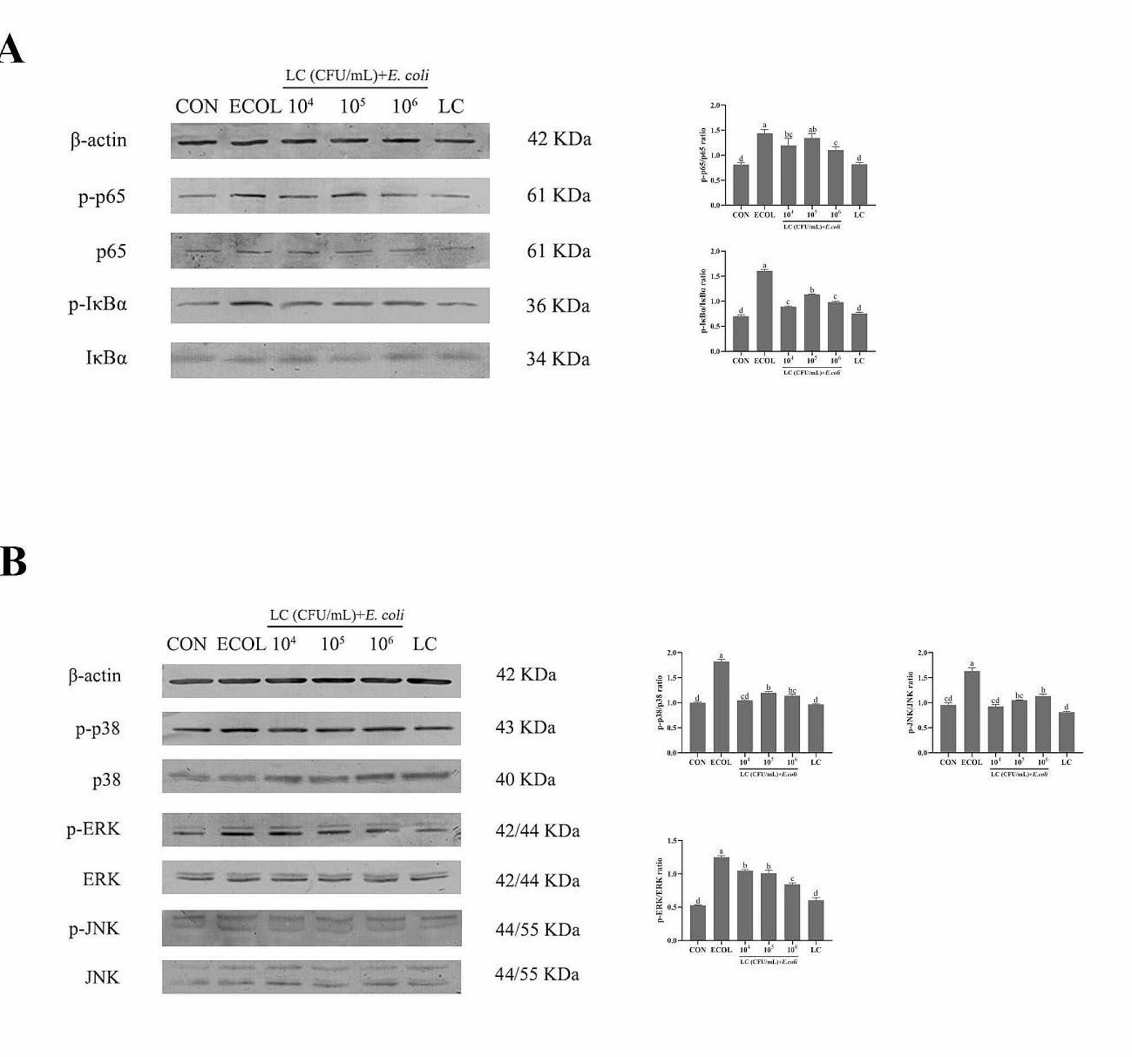
Effects of *L. casei* 03 on protein expression involving in NF-κB (**A**) and MAPK (**B**) Signaling Pathways in *E. coli*-induced BMECs. β-actin was used as a control. Values were presented as mean ± SEM from three independent experiments

We further evaluated the effects of *L. casei* 03 on the activity of the NF-κB and MAPK signaling pathways by Western Blot. As shown in Fig. 4A and B, the phosphorylation levels of p65 and IκBα in the NF-κB signaling pathway and the phosphorylation levels of p38, ERK and JNK in the MAPK signaling pathway were significantly increased in the ECOL group. However, all of them were significantly decreased after pretreatment with *L. casei* 03 (*P* < 0.05). Additionally, there were no significant changes in these protein expressions in the LC group compared with the CON group.

### Effects of *L. casei* 03 on mammary gland tissue histopathological changes in *E. coli*-induced mastitis

**Fig. 5:**
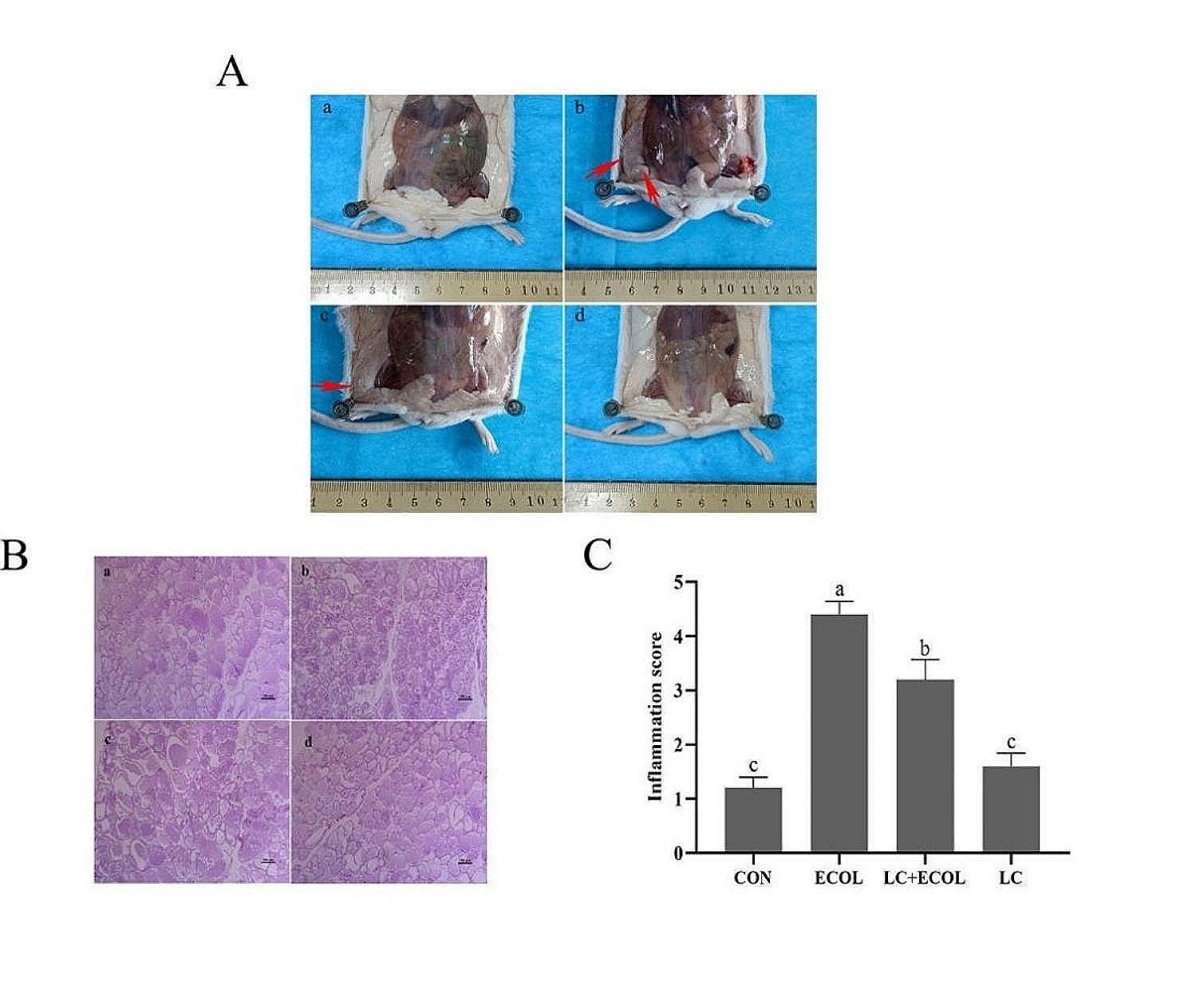
A The morphology of the mammary glands from CON group (**a**), ECOL group (**b**), LC + ECOL group (**c**) and LC group (**d**). B The histological characteristics of mice mammary tissue in *E. coli*-induced Mastitis. Representative images from CON group (**a**), ECOL group (**b**), LC + ECOL group (**c**) and LC group (**d**). scale bar: 200 μm. C The inflammation score of representative images from each group. Scores range from 1 to 5, with higher scores indicating more severe inflammatory change. Values were presented as mean ± SEM (*n* = 5)

Various degrees of redness, bleeding or swelling was seen in the mammary tissues in the ECOL and LC + ECOL groups. In parallel, there were no apparent histopathologic changes in the CON and LC groups (Fig. 5A). The histological characteristics of mice mammary tissue were assessed by H&E staining (Fig. 5B). There were no obvious inflammatory changes in mice mammary tissue from the CON and LC groups. In contrast, the mammary tissue in the ECOL group presented serious histopathological changes, such as thickened alveolar walls, edema, hyperemia and inflammatory cell infiltration. However, these pathological changes were ameliorated by *L. casei* 03 pretreatment. The same conclusion was obtained in the inflammation score (Fig. 5C). Pretreatment with *L. casei* 03 significantly reduced the score rise induced by *E. coli* in mice mammary tissue (*P* < 0.05).

### Effects of *L. casei* 03 on MPO activity in mice mammary tissue

**Fig. 6:**
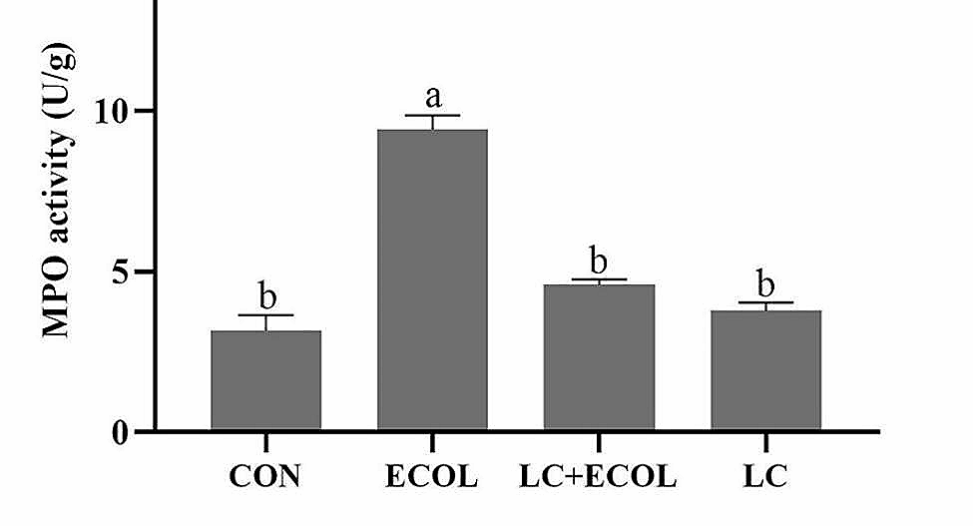
MPO activity in the homogenate of mice mammary tissues from the control group, *E. coli* group, pretreatment with *L. casei* 03 and *L. casei* 03 alone group. Data were presented as mean ± SEM (*n* = 4)

The MPO activity in mice mammary tissues from each group was shown in Fig. 6. The results showed that *E. coli* challenge significantly increased MPO activity in mammary tissue compared with that in the CON group (*P* < 0.05). However, these elevations were inhibited after pretreatment with *L. casei* 03.

### Effects of *L. casei* 03 on proinflammatory cytokine levels in mice mammary tissue

**Fig. 7:**
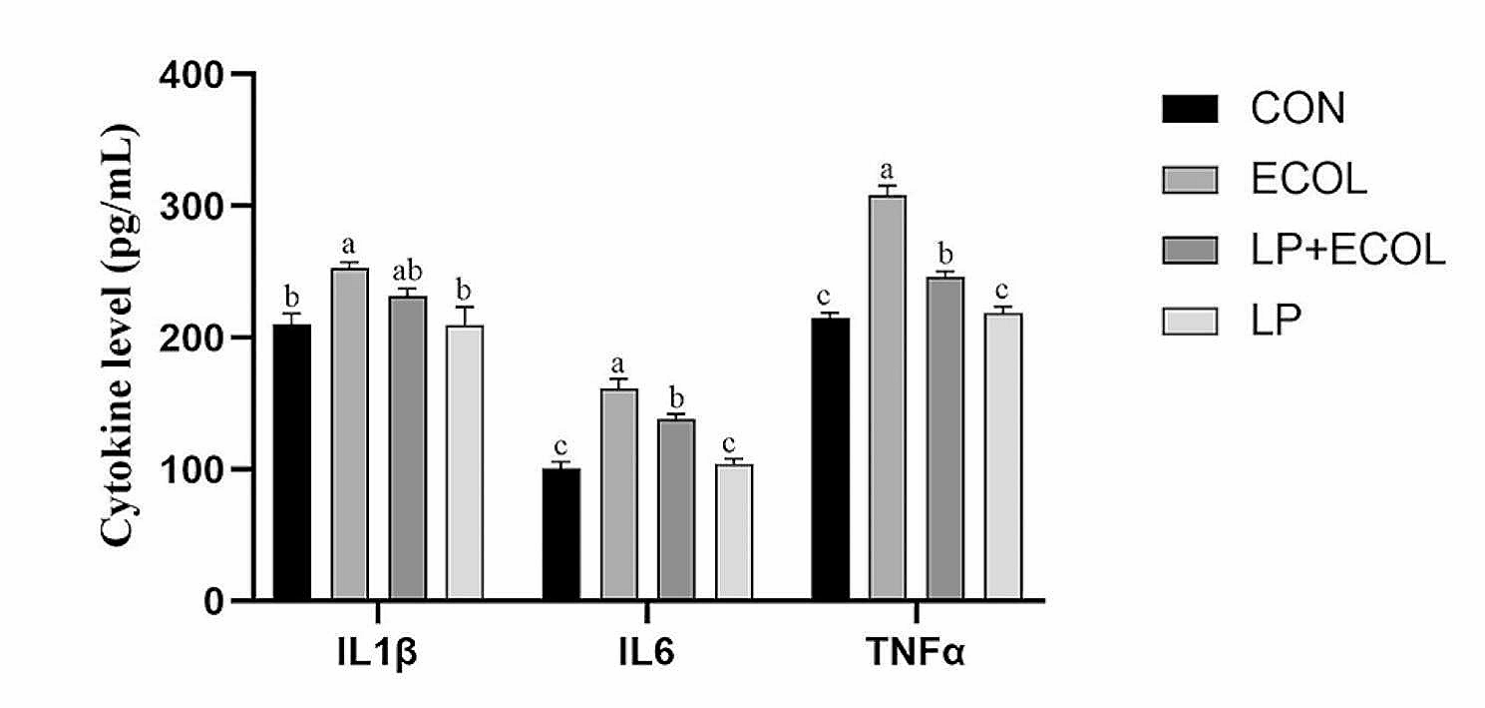
The levels of IL1β (**A**), IL6 (**B**) and TNFα (**C**) in the homogenate of mice mammary tissues including the control group, *E. coli* group, and pretreatment with *L. casei* 03 and *L. casei* 03 alone groups. Data expressed as mean ± SEM (*n* = 4)

The secretion levels of IL1β, IL6 and TNFα in mice mammary tissues were detected by ELISA. As shown in Fig. 7, after *E. coli* treatment, the levels of IL1β, IL6 and TNFα in mammary tissue increased significantly compared with those in the CON group (*P* < 0.05). Pretreatment with *L. casei* 03 suppressed these increases by varying degrees.

## Discussion

Dairy cow mastitis has always been a focus of veterinary personnel and is a major problem that hinders earnings growth in dairy farms [16]. Antibiotics, the most commonly used method to control this disease, obtain a good clinical response while also leading to the emergence of multidrug-resistant (**MDR**) bacteria [17]. MDR bacteria not only increase the difficulty of treating disease but brings a potential threat to human health [18]. Therefore, Therefore, seeking effective alternative antimicrobial agents for mastitis is urgent and essential. *E. coli* is a major pathogen responsible for clinical mastitis in dairy cows, which usually causes severe inflammatory reactions and economic losses [19, 20]. Recent studies show that some lactic acid bacteria exert direct suppressive effects on *E. coli* isolated from dairy cow mastitis and avoid the emergence of resistant bacteria. These properties provide an excellent rationale for alternative antibiotic strategies in mastitis [21, 22]. Therefore, we screened several strains of probiotics based on previous trials and *L. casei* 03 gradually entered our field of vision with its potential anti-inflammatory properties.

Mammary epithelial cells form an important component of milk synthesis in mammary tissue and are involved in the local innate immune responses [23]. When pathogens gain access to mammary tissues, many pathogens associated molecular patterns are recognized by the Toll-like receptor (**TLR**) of surface proteins on cells, which in turn can activate intracellular signaling pathways and promote the expression of the other inflammatory cytokines and enlarges the inflammatory response [24, 25]. It is widely accepted that overproduction of these inflammatory mediators and cytokines is highly associated with inflammatory diseases [26]. IL6, TNFα and IL1β are the common pro-inflammatory cytokines, they can play a cytotoxic role in the inflammatory process and accelerate the inflammatory processes [27]. In this study, we first used in vitro inflammatory models established with *E. coli*-treated BMECs to simulate the pathogenesis of mastitis. The results showed that *L. casei* 03 pretreatment reduced the elevated mRNA expression of pro-inflammatory genes caused by *E. coli*. This suggests that *L. casei* 03 can exert an inflammatory ameliorating effect by inhibiting the expression of pro-inflammatory factors. Many studies have shown that NF-κB and MAPK pathways play key roles in inflammation modulation [28–30]. We next investigated the effect of *L. casei* 03 on the activity of NF-κB and MAPK signaling pathways. Our results showed that *L. casei* 03 could inhibit the elevated phosphorylation levels of key pathway proteins in signaling pathways such as p65 and p38 caused by *E. coli* and exert anti-inflammatory effects via inhibiting NF-κB and MAPK activity, this matches the result of Zheng et al. [31]. Apoptosis is inextricably linked with excessive inflammatory cascades [32]. Some endotoxins in *E. coli* can trigger inflammatory responses and induce cell apoptosis. Meanwhile, apoptotic cells release cytokines and other inflammatory mediators, thereby exacerbating inflammation [33]. Previous studies showed that *E. coli* treatment increased the apoptosis rate in bovine endometrial epithelial cells, and *Lactobacillus rhamnosus* can inhibit this increase [34]. Similarly, our study also showed that *L. casei* 03 could inhibit *E. coli*-induced apoptosis in BMECs and had positive effects on anti-inflammation.

Based on the anti-inflammatory effects of *L. casei* 03 in vitro, we next investigated the protective effects of *L. casei* 03 in *E. coli*-induced mastitis in the mouse model. The characteristics of mastitis mainly manifest as mammary swelling, bleeding, inflammatory cell infiltration and acinar injury [35, 36]. Simultaneously, MPO activity in tissue is increased due to BMEC damage [37]. After injecting *E. coli* into mice mammary tissue, we observed a series of histopathological changes, as well as a significantly increased pro-inflammatory factor and MPO activity in mammary tissue. These inflammatory symptoms were significantly alleviated with *L. casei* 03 pretreatment. These results indicate that *L. casei* 03 has protective effects on *E. coli*-induced mastitis in vivo.

Notably, some academics have expressed concerns about the safety of the application of probiotics treatment in mastitis, noting that intramammary infusion with live probiotics can cause or aggravate mastitis [38]. However, some scholarly studies dispelled this concern to some extent. Martín et al. successfully isolated probiotics from healthy breast tissue to demonstrate the presence of probiotics in normal mammary tissue [39]. Zheng et al. also showed the feasibility of intramammary infusion of *L. casei* in mastitis treatment [31]. In this study, we investigated the effect of *L. casei* 03 on the viability of BMECs in vitro and selected safe doses for subsequent experiments. We subsequently set up the *L. casei* 03 alone treatment group and did not detect appreciable toxic effects in vitro models. This demonstrates the application safety and reliability of *L. casei* 03 and provides the basis for its subsequent use in mastitis treatment.

## Conclusions

In conclusion, *L. casei* 03 could inhibit the NF-κB and MAPK signaling pathway activity and reduce the pro-inflammatory gene expression to resist the inflammatory response induced by *E. coli* in BMECs. The in vivo results suggested that *L. casei* 03 alleviated pathological changes and pro-inflammatory factors secretion in mammary tissue and exerted anti-inflammatory effects in *E. coli*-induced mice mastitis. The present study demonstrates that *L. casei* 03 has preventive effects on *E. coli*-induced mastitis in vitro and in vivo and this provides new insights for the prevention and treatment of dairy cow mastitis.

### Electronic supplementary material

Below is the link to the electronic supplementary material.

The original, full length blots of western blot

## Data Availability

All data used to support the findings in this study are included within the article. The original, full-length western blot blots are listed in the supplementary information.

## Abbreviations

BMECs: Bcovine mammary epithelial cells
FITC: Fluorescein isothiocyanate
MPO: Myeloperoxidase
NF-κB: Nuclear factor kappa B
MAPK: Mitogen-activated protein kinase
MDR: Multidrug-resistant
TLR: Toll-like receptor
