# Supplementary figures and images for: The preventive effects of Lactobacillus casei 03 on Escherichia coli-induced mastitis in vitro and in vivo
Source: J Inflamm (Lond). 2024 Feb 23;21:5. doi: 10.1186/s12950-024-00378-x (PMC10893599; doi:10.1186/s12950-024-00378-x)

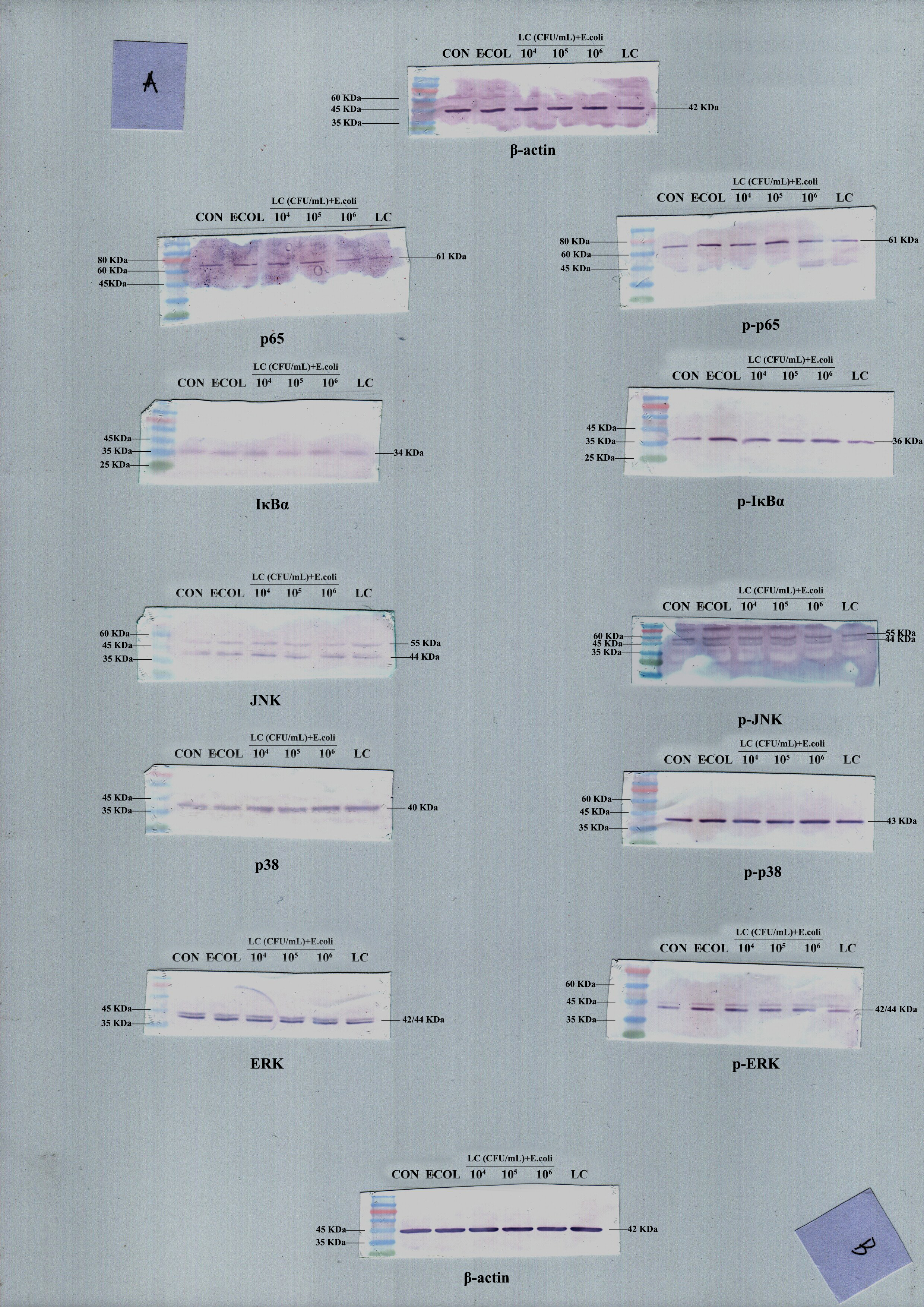

Supplement: Supplementary file 1 — The original, full length blots of western blot [file 12950_2024_378_MOESM1_ESM.jpg]
